# Quality of Life, Depression, and Healthcare Resource Utilization among Adults with Type 2 Diabetes Mellitus and Concomitant Hypertension and Obesity: A Prospective Survey

**DOI:** 10.1155/2012/404107

**Published:** 2012-06-17

**Authors:** Andrew J. Green, Debbra D. Bazata, Kathleen M. Fox, Susan Grandy

**Affiliations:** ^1^Midwestern Endocrinology, Overland Park, KS 66211, USA; ^2^Saint Luke's South Primary Care, Overland Park, KS 66213, USA; ^3^Strategic Healthcare Solutions, LLC, P.O. Box 543, Monkton, MD 21111, USA; ^4^AstraZeneca, Wilmington, DE 19803-2902, USA

## Abstract

*Background*. This study compared quality of life, depression, and healthcare resource utilization among adults with type 2 diabetes mellitus (T2DM) and comorbid hypertension (HTN) and obesity with those of adults reporting T2DM alone. *Methods*. Respondents to the US SHIELD survey self-reported their height, weight, comorbid conditions, hospitalizations, and outpatient visits and completed the Short Form-12 (SF-12) and Patient Health Questionnaire (PHQ-9). Respondents reporting T2DM and HTN and obesity (body mass index, BMI, ≥30 kg/m^2^) were compared with a T2DM-alone group. *Results*. Respondents with T2DM, HTN, and obesity (*n* = 1292) had significantly lower SF-12 Physical and Mental Component Summary scores (37.3 and 50.9, resp.) than T2DM-alone respondents (*n* = 349) (45.8 and 53.5, resp., *P* < 0.0001). Mean PHQ-9 scores were significantly higher among T2DM respondents with comorbid HTN and obesity (5.0 versus 2.5, *P* < 0.0001), indicating greater depression burden. Respondents with T2DM, HTN, and obesity had significantly more resource utilization with respect to physician visits and emergency room visits but not hospitalizations than respondents with T2DM alone (*P* = 0.03). *Conclusions*. SHIELD respondents with comorbid conditions of T2DM, HTN, and obesity reported greater healthcare resource utilization, more depression symptoms, and lower quality of life than the T2DM-alone group.

## 1. Background

Adults with type 2 diabetes mellitus (T2DM) are likely to have other health conditions that may adversely impact their health status and glycemic control [1, 2]. Hypertension (HTN), obesity, hyperlipidemia, and cigarette smoking act as independent modifiable contributors to cardiovascular disease (CVD) in patients with diabetes [3]. Current American Diabetes Association and International Diabetes Federation standards of medical care aim to reduce the vascular complications through control of blood glucose, blood pressure, and blood lipids [4, 5].

Individuals with T2DM are known to have lower quality of life and more depressive symptomatology than those without diabetes [4–6], yet the impact may be partially due to comorbid conditions. Additionally, adults with diabetes have been shown to be frequent consumers of healthcare resources for routine physician visits, eye and foot examinations, monitoring of therapy, and management of glucose and other comorbid conditions [7, 8]. Thus, it would be hypothesized that individuals with T2DM and comorbid conditions of HTN and obesity would have a greater negative impact on quality of life, greater burden of depression, and greater use of healthcare resources if these conditions were not monitored and treated. Notwithstanding, little information is available regarding health outcomes among T2DM adults with comorbid conditions of HTN and obesity who are treated in the community. The present study, one of the first investigations, was implemented to assess quality of life, depression, and healthcare resource utilization among adults with self-reported T2DM and comorbid HTN and obesity, a high-risk group, compared with those among adults reporting T2DM alone.

## 2. Methods

The present investigation is a cross-sectional analysis of data from the Study to Help Improve Early evaluation and management of risk factors Leading to Diabetes (SHIELD) comparing health outcomes (health-related quality of life (HRQOL), depression, and healthcare resource utilization) between respondents with T2DM and comorbid HTN and obesity and respondents with T2DM alone. SHIELD is a 5-year, survey-based study conducted to better understand patterns of health status, health behavior, and knowledge and attitudes of people living with diabetes and those with varying levels of cardiometabolic risk.

### 2.1. SHIELD Survey

SHIELD included an initial screening phase to identify cases of interest in the general population (e.g., diabetes mellitus), a baseline survey to follow up identified cases with a questionnaire about health status, health knowledge and attitudes, and current behaviors and treatments, and annual follow-up surveys. A detailed description of the SHIELD methodology has been published previously [9, 10].

In brief, the screening survey was mailed in April 2004 to a stratified random sample of 200,000 US households, representative of the US population for geographic residence, household size and income, and age of head of household [11], identified by the Taylor Nelson Sofres National Family Opinion (TNS NFO) panel (Greenwich, CT). All TNS NFO surveys were voluntary, and no special incentives were provided. A response rate of 64% (127,420 households representing 211,097 individuals) was obtained for the screening survey. The SHIELD study was approved by the Quorum Review Board.

A comprehensive baseline survey was mailed in September-October 2004 to a representative sample of individuals, 18 years or older (*n* = 22,001), who were identified in the screening survey as having self-reported T2DM or type 1 diabetes mellitus, no diabetes, or being at risk for diabetes. Each respondent group was balanced to be representative of that segment of the population for age, gender, geographic region, household size, and income for the US population, and then a random sample from each group was selected and sent the baseline survey. A response rate of 72% was obtained for the baseline survey. The 2008 annual follow-up survey collected information from 14,921 SHIELD respondents (response rate of 71%) to identify those with the triad conditions of T2DM, HTN, and obesity. Responses from the 2008 survey were analyzed and reported in this study.

### 2.2. Study Measures

Respondents were classified as having T2DM based upon their self-report of having been told by a doctor, nurse, or other healthcare professional that they had T2DM. Among the T2DM respondents, two cohorts were identified: (1) those reporting comorbid HTN plus obesity and (2) those without a self-report of HTN and obesity. Respondents were classified as having HTN based on their self-report of having been told by a healthcare professional that they had high blood pressure/HTN. Obesity was defined as a body mass index (BMI) ≥30 kg/m^2^.

The Short Form-12 version 2 (SF-12), a validated scale, was used to assess HRQOL. The SF-12, the short version of the widely used SF-36, is a brief and reliable generic measure of overall health status [12]. The SF-12 measures 8 domains of health: physical functioning, role limitations because of physical health, bodily pain, general health perceptions, vitality, social functioning, role limitations because of emotional problems, and mental health. The recall period was the past 4 weeks. SF-12 responses were scored from 0 to 100 on the Physical Component Summary (PCS) scale and Mental Component Summary (MCS) scale. Higher scores indicate better HRQOL. To simplify comparisons with the general population, norm-based scoring was used. In norm-based scoring, scores are linearly transformed to a scale with a mean of 50 and standard deviation (SD) of 10 for the general population [12].

The Patient Health Questionnaire (PHQ-9) focuses on the nine signs and symptoms of depression from the Diagnostic and Statistical Manual of Mental Disorders, Fourth edition (DSM-IV) [13]. The PHQ-9 is a dual-purpose, validated instrument that is used to establish a provisional depressive disorder diagnosis as well as provide a symptom severity score. Higher scores indicate increasing severity of depression. For a diagnosis of depression, five or more items must be scored as present more than half of the days or nearly every day. A PHQ-9 score of 5–9 indicates minimal depressive symptoms, a score of 10–14 is minor depression or major depression that is mild, a score of 15–19 is major depression, moderately severe, and a score ≥20 indicates major depression, severe [13].

For healthcare resource utilization, respondents reported the number of times or number of days in the past 12 months they visited or stayed overnight for each type of health facility due to their health problems. The health facilities included hospitals, emergency room or urgent care facility, and physician office/clinic.

### 2.3. Statistical Analysis

Comparisons between T2DM respondents with and without comorbid conditions of HTN and obesity were conducted using chi-square test for categorical variables and *t*-tests for continuous variables. Statistical significance was set *a priori* as *P* < 0.05.

## 3. Results

A total of 1,395 respondents reported T2DM and comorbid conditions of HTN and obesity (triad conditions), and 370 reported T2DM alone. An additional 680 respondents reported T2DM and HTN only, and 440 had T2DM and obesity only; these respondents were not included in the analysis. Respondents with the triad conditions were younger, more often women, more nonwhite, and had higher annual household income than respondents with T2DM alone (*P* < 0.001, Table 1). Respondents with the triad conditions were similar to respondents with T2DM alone in education and cardiovascular disease history. Diabetes drug therapy was similar between the groups, with 63% of each group receiving oral antidiabetic drugs alone and 8% receiving insulin alone. Approximately 92% of the respondents with the triad conditions received antihypertensive therapy.

### 3.1. Health-Related Quality of Life

A small proportion of respondents did not complete the SF-12; 103 (7.4%) respondents with T2DM and comorbid HTN and obesity and 21 (5.7%) respondents with T2DM alone. Among those responding, respondents with T2DM, HTN, and obesity had significantly lower PCS and MCS scores (37.3 and 50.9, resp.) than respondents with T2DM alone (45.8 and 53.5, resp.) (Table 2). Physical health scores were below the norm (<50.0) of the general population for both respondents with the triad conditions and those with T2DM alone.

### 3.2. Depression

Mean PHQ-9 scores were significantly higher among T2DM respondents with comorbid HTN and obesity (5.0 versus 2.5) than among respondents with T2DM alone (*P* < 0.001). A significantly greater proportion of respondents with T2DM and comorbid HTN and obesity had mild-to-severe depression based on the PHQ-9 scores (*P* < 0.001) (Table 2). Additionally, 16.5% of respondents with T2DM, HTN, and obesity had moderate-to-severe depression, compared with 6.1% of respondents with T2DM alone (*P* < 0.001).

### 3.3. Healthcare Resource Utilization

**Figure 1:**
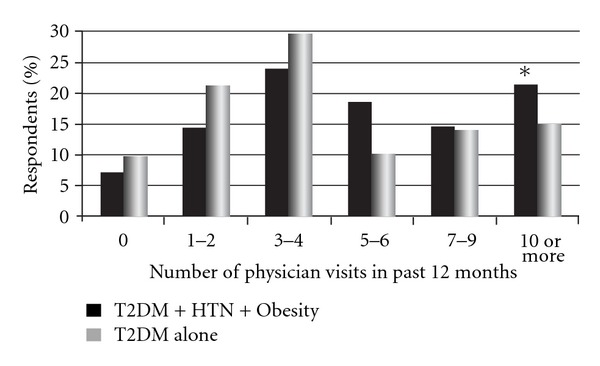
Number of physician visits in the past 12 months for T2DM respondents with and without comorbid conditions of hypertension and obesity.

Respondents with T2DM, HTN, and obesity had significantly more physician office visits (mean = 7.7 versus 6.0) than respondents with T2DM alone (*P* = 0.001) (Figure 1). More respondents with the triad conditions had 10 or more physician visits in the past 12 months than respondents with T2DM alone (*P* = 0.03); moreover, 5.7% of respondents with the triad conditions versus 1.9% of T2DM-alone respondents had 20 or more physician visits. Approximately 54% of respondents with T2DM, HTN, and obesity visited only their primary care physician (PCP) in the past 12 months, whereas 15% visited their PCP and cardiologist, 19% visited their PCP and endocrinologist, and 12% visited their PCP, cardiologist, and endocrinologist. There was no difference in the type of physician seen between the two T2DM groups.

Respondents with T2DM, HTN, and obesity reported significantly more emergency room visits (mean = 1.9 versus 1.4) than respondents with T2DM alone (*P* = 0.02) (Table 3). More respondents with the triad conditions had 2 or more emergency room visits (9%) than respondents with T2DM alone (5%, *P* = 0.02). A similar proportion of respondents (20%) reported being hospitalized at least once in the past 12 months between groups (*P* > 0.05). There was no significant difference between groups in the number of days that respondents were hospitalized (*P* = 0.15).

## 4. Discussion

SHIELD respondents with comorbid conditions of T2DM, HTN, and obesity reported lower HRQOL, both physical and mental health, and greater burden from depression than respondents with T2DM alone. Healthcare resource utilization was substantial among respondents with T2DM, HTN, and obesity. The number of physician visits and emergency room visits was significantly greater among respondents with the triad conditions, compared with T2DM-alone respondents. Even though physician visits were frequent among respondents with the triad conditions, less than 50% reported seeing a cardiologist or endocrinologist over the past 12 months. The healthcare visits may be an opportunity for patients and physicians to communicate, evaluate, and better manage chronic disease conditions such as diabetes, HTN, and obesity.

This study demonstrated worse health outcomes for respondents with T2DM, HTN, and obesity, but it is unknown in which way these three conditions interrelate with each other and with the health outcomes. It is unclear which event/outcome comes first. It is possible that having multiple health problems (T2DM, HTN, and obesity) is overwhelming, leading to lower HRQOL and depression. Further research is needed to determine if it is a vicious circle of events or if the disease leads to lower HRQOL and depression or if lower HRQOL and depression lead to the development of T2DM, HTN, and obesity.

Previous studies have clearly established that adults with T2DM have reduced quality of life and greater depression compared with the general population [4–6]. However, this is one of the first studies to identify a group of T2DM, that is, those with T2DM, HTN, and obesity, who have lower quality of life and greater depression than those with T2DM alone. It is important for healthcare providers to identify their T2DM patients with the triad conditions, and manage all 3 conditions to reduce the potential for lower quality of life and depression, which may result in poorer self-management of health and continued progression of disease. The frequent healthcare visits to physicians provide individuals and their physicians an opportunity for disease management that can lead to better health outcomes. Each physician visit should be viewed as a venue for monitoring and reinforcing glycemic control, blood pressure control, and weight management. A previous analysis of the SHIELD data indicated that T2DM respondents who reported exercising regularly had higher physical and mental quality of life than those who did not exercise regularly, and that those T2DM respondents who tried to lose weight had higher mental quality of life than those who did not try to lose weight [14]. Thus, if each physician visit was utilized as an opportunity to promote regular exercise and weight management, and patients incorporated exercise and weight management into their daily routine, then quality of life may improve for individuals with the triad conditions.

One of the strengths of the study is that validated instruments (SF-12 and PHQ-9) have been used to measure the health outcomes (HRQOL and depression) of individuals with T2DM being treated in the community. The SF-12 and PHQ-9 are standardized, validated instruments that have been used in numerous studies with diverse populations.

This study has some limitations that need to be considered in the interpretation of the results. The diagnosis of diabetes, HTN, other comorbid conditions, healthcare resource utilization, and weight were self-reported and could not be validated with medical record review or administrative claims data. However, this bias is similar between the two groups compared in this study. Household panels, like the SHIELD study, tend to underrepresent the very wealthy and very poor segments of the population and do not include military or institutionalized individuals. HbA1c levels were not collected in the SHIELD survey; therefore, no information is available for diabetes control for the groups. Differences between groups in demographic composition (i.e., more women and higher income in the triad group) may have been associated with increased depression and healthcare resource utilization for the triad group since these factors were not controlled for in this observational, real-world outcomes study.

In conclusion, individuals with T2DM and the comorbid conditions of HTN and obesity reported lower HRQOL and greater depression burden than adults with T2DM alone. These individuals with the triad conditions also had more physician visits and emergency room visits than those with T2DM alone. Each contact with the healthcare system could be an opportunity to better manage all 3 conditions to reduce the negative impact on quality of life. Further research is needed to ascertain whether poor quality of life and greater depression in respondents with T2DM, HTN, and obesity affect self-management of their diabetes and comorbid conditions.

## Figures and Tables

**Table 1:** Characteristics of SHIELD respondents with triad conditions versus T2DM alone.

Characteristics	T2DM + HTN + Obesity *N* = 1,395	T2DM alone *N* = 370
Age, years, mean (SD)	61.4 (10.8)^∗^	66.2 (12.9)
Women, %	65.4^∗^	45.9
White, %	72.5^∗^	76.8
Education, % with at least some college	65.6	66.7
Income, % with ≥$30,000/year	60.6^∗^	72.7
Dyslipidemia, %	79.4^∗^	56.9
Heart disease/heart attack, %	24.4	19.8
Stroke or TIA, %	5.5	5.7

^∗^ *P* < 0.01.

HTN: hypertension; SD: standard deviation; T2DM: type 2 diabetes mellitus; TIA: transient ischemic attack.

**Table 2:** SF-12 physical and mental component summary scores and PHQ-9 depression scores for T2DM respondents with and without comorbid hypertension and obesity.

	T2DM + HTN + Obesity *N* = 1,292	T2DM alone *N* = 349
SF-12		
Physical component summary score	37.3 (12.6)^∗^	45.8 (11.7)
Mental component summary score	50.9 (11.0)^∗^	53.5 (9.3)
PHQ-9		
Summary score, mean (SD)	5.0 (5.6)^∗^	2.5 (4.4)
No depression, % with score 0–4	59.8	82.8
Minimal depression, % with score 5–9	23.7	11.0
Mild depression, % with score 10–14	8.4	2.0
Moderately severe depression, % with score 15–19	4.7	2.6
Major depression, severe, % with score ≥20	3.4	1.5

^∗^ *P* < 0.001.

HTN: hypertension; SD: standard deviation; T2DM: type 2 diabetes mellitus.

**Table 3:** Healthcare resource utilization for T2DM respondents with and without comorbid hypertension and obesity.

Resource use	T2DM + HTN + Obesity *N* = 1,395	T2DM alone *N* = 370
Emergency room visits		
0 visits, %	80.3	82.4
1 visit, %	11.3	12.4
2 or more visits, %	8.4	5.1
Mean (SD) for those with ≥1 visit	1.9 (1.5)^∗^	1.4 (0.8)
Hospitalizations		
≥1 hospital stay in past 12 months	20.9	20.1
Median (IQR) total number of days hospitalized (all stays) for those with ≥1 hospital stay	4.0 (2–8)	3.0 (2–10)

^∗^ *P* = 0.02.

HTN: hypertension; SD: standard deviation; T2DM: type 2 diabetes mellitus; IQR: interquartile range.

## Abbreviations

BMI: Body mass index
HRQOL: Health-related quality of life
HTN: Hypertension
MCS: Mental component summary
PCP: Primary care physician
PCS: Physical component summary
PHQ-9: Patient health questionnaire
SF-12: Short Form 12 items
SHIELD: Study to Help Improve Early evaluation and management of risk factors Leading to Diabetes
T2DM: Type 2 diabetes mellitus
TNS NFO: Taylor Nelson Sofres National Family Opinion.
